# Distinct Transcriptional and Migratory Programs Are Associated with Vasculogenic Mimicry Heterogeneity in Triple-Negative Breast Cancer

**DOI:** 10.3390/cancers18111789

**Published:** 2026-05-29

**Authors:** Shilpa Madhavan-Kadali, Hyun-Mi Cho, Tal Sneh, Naamah Bloch, Joseph D. Rosenblatt, Abraham O. Samson, Hava Gil-Henn

**Affiliations:** 1 The Azrieli Faculty of Medicine, Bar-Ilan University, Henrietta Szold Street 8, Safed 1311502, Israel; sashacasper5@gmail.com (S.M.-K.); naamah.bloch@biu.ac.il (N.B.); 2 Department of Medicine, Division of Hematology, Sylvester Comprehensive Cancer Centre, University of Miami Miller School of Medicine, Miami, FL 33136, USA

**Keywords:** vasculogenic mimicry, triple-negative breast cancer, TNBC, endothelial angiogenesis, tube formation assay, DepMap, cancer stem cell (CSC), epithelial–mesenchymal transition (EMT), extracellular matrix (ECM) remodeling

## Abstract

Background: Vasculogenic mimicry (VM) is a tumor-driven vascularization strategy in which aggressive cancer cells form perfusable, endothelium-independent channels that support tumor growth, metastasis, and therapy resistance. VM is prevalent in triple-negative breast cancer (TNBC), but within this group of tumors, VM heterogeneity is underexplored. Likewise, VM competence and its relationship to classical endothelial angiogenesis (EA) remain incompletely understood. Methods: Here, as a proof of concept, we combine functional analysis of three molecularly distinct TNBC cell lines with a panel-wide DepMap transcriptomic survey to characterize VM heterogeneity. Results: Using an in vitro tube formation assay, we show that the VM-competent TNBC cell lines MDA-MB-231 and MDA-MB-231-4175 form robust 3D vessel-like networks in a matrigel matrix, whereas the VM-incompetent line MDA-MB-468 does not. As a control, we use an immortalized endothelial cell line, 3B-11, that forms classical EA vessel-like networks. Moreover, we visualize VM (Laminin-5^+^) and EA (CD31^+^) markers in vessel-like networks of VM-competent TNBC xenografts using immunohistochemical staining and show that while they are distinctly labeled, they can also coexist to form mosaic-like vessels. Then, we use DepMap-based transcription profiles and reveal that VM competence is associated with a distinct signature. Interestingly, VM and EA transcription profiles partially overlap, yet they also remain transcriptionally distinct, with inferred mechanistic divergence, with VM being more associated with cancer cell stemness (CSC), epithelial-to-mesenchymal transition (EMT), and extracellular matrix (ECM) remodeling programs and EA being more associated with vessel strength. In addition, VM-competent TNBC cells display migration patterns and transcriptomic features consistent with endothelial-like mechanosensitivity. Conclusions: Together, these findings indicate that VM is a distinct, heterogeneous, and therapy-relevant state in TNBC that complements classical angiogenesis. Finally, the mechanistic distinction between VM and EA programs made here will motivate future studies on dual-targeting strategies that inhibit both vascularization processes while also motivating future studies on VM for precision treatment in TNBC.

## Simple Summary

Cancerous tumors need blood to grow and spread. Most tumors do this by recruiting blood vessels from surrounding tissue, but some aggressive cancers can build their own vessel-like channels directly from tumor cells, which conventional drugs fail to block. In this study of triple-negative breast cancer, a particularly aggressive form of the disease, we found that the ability to build these tumor channels varies between tumor cells, and we identified cellular features that distinguish them, providing a foundation for new therapies.

## 1. Introduction

Vasculogenic mimicry (VM) is a process where highly aggressive tumor cells self-assemble into perfusable vessel-like structures that support cancer growth and metastasis. VM channels conduct fluids and nutrients to the tumor and complement classical EA as a vascular supply [1]. Unlike conventional blood vessels, which are lined by endothelial cells, VM channels are formed by tumor cells and lack an endothelial lining [1]. VM was first described in a highly invasive and malignant melanoma by Maniotis et al. [2]. Despite the initial skepticism, VM has since been confirmed to take place across numerous aggressive malignancies, such as breast cancer [3,4], pancreatic cancer [5,6], non-small-cell lung cancer [7,8], hepatocellular carcinoma [9,10], and glioblastomas [1,11]. Recently, VM has been found in triple-negative breast cancer cells [12,13]. TNBC is an aggressive, fast-growing highly invasive cancer that tests negative for estrogen receptor (ER), progesterone receptor (PR), and human epidermal growth factor 2 (HER2) [14]. It is characterized by rapid tumor progression, a high risk of metastasis, repeated relapse and poor survival rates. Treatment options remain limited due to the lack of targetable receptor expression, and standard therapy is largely restricted to chemotherapy [15]. Notably, TNBC exhibits a higher prevalence of VM than other breast cancers, and TNBC is a good model for VM studies [16]. These discoveries represent a major shift in tumor vascular biology, challenging the long-standing view that EA constitutes the tumors’ sole vascular lifeline.

EA is a key hallmark of tumor growth, supplying cells with blood, nutrients, oxygen, and a primary route of metastatic diffusion [17,18]. However, EA differs from VM in more ways. For example, anti-angiogenic therapy (AAT) inhibits EA but has little or no effect on VM. In some cases, AAT may even promote VM as a compensatory mechanism, allowing tumors to maintain perfusion and survive despite angiogenic blockade. This highlights that VM is a stable and therapy-resistant vascular structure within tumors [19,20,21]. Moreover, mosaic vessels, structures composed of both endothelial and tumor cells, are frequently observed in aggressive tumors, suggesting a functional interplay between EA and VM [22]. VM also exhibits functional and structural heterogeneity across patients with the same cancer (inter-tumor heterogeneity) and within individual tumors (intra-tumor heterogeneity) [16]. Although EA and VM can produce structurally similar vessel-like conduits, fundamental molecular differences distinguish them from each other. EA vessels typically express endothelial markers such as CD31^+^ and CD34^+^, whereas VM channels generally lack endothelial markers and instead exhibit Laminin-5^+^ basement membrane structures deposited by tumor cells themselves. Currently, the extent of transcription overlaps between EA and VM, the degree of co-option of endothelial pathways, and their role as a distinct tumor-intrinsic mechanism remain an area under investigation.

The transcriptional signature of VM has been studied extensively, and it is closely guided by EMT [23]. EMT is a key prerequisite in which cancer cells lose epithelial integrity and acquire endothelial-like cell characteristics to self-assemble into endothelium-independent vasculatures [24]. EMT induces endothelial-like behavior, such as migratory capacity, invasiveness, and ECM remodeling. EMT is driven by hypoxia, and it is mediated by upregulation of HIF-1α. HIF-1α subsequently activates several critical downstream interactors—such as VEGF, EGFR, TWIST1, SLUG, ZEB1, SNAIL, vimentin, and N-cadherin—that promote VM formation [25]. Interestingly, a hypoxic microenvironment, together with increasing ECM stiffness, promotes sprouting of endothelium-dependent vasculatures. ECM stiffness induces a distinct VM-like morphology in metastatic lung and breast cancer cells [26], and matrix rigidity is translated into mechanosensitive transcriptional programs that support vascular-like remodeling [27]. In addition, hypoxia upregulates the expression of matrix metalloproteases such as MMP2 and MMP9 in cancer cells, facilitating the deposition of laminin-5 γ2, an ECM component that contributes to VM formation [28,29]. Furthermore, EMT-activating transcription factors such as TWIST, ZEB, and SNAIL promote the acquisition of a CSC-like phenotype through activation of pluripotency regulators, including SOX2, OCT4, and NANOG [30]. Recent studies suggest that the CSCs resulting from EMT represent a form of stress-induced transient cell plasticity. These cells are thought to line VM channels and sustain their development by supporting the expression of VM-specific molecules [31,32]. Nevertheless, the transcriptional signature of VM across different TNBCs is relatively unknown.

In this study, we explore VM in three TNBC lines, namely, MDA-MB-231, MDA-MB-231-4175, and MDA-MB-468, using in vitro tube formation and 3B-11 endothelial cells as the EA control. We then extend the analysis transcriptomically across DepMap (71 breast cancer lines with ABMT0725 as a human endothelial reference) and the TCGA-BRCA, METABRIC, and GSE111653 datasets. Using this data, we the signature overlap between the CSC, EMT, ECM, and mechanosensitive migration in channel formation. Our findings suggest that while VM and EA partially overlap, they also differ in key aspects, such as molecular profiles and key programs supporting vessel identity. Our findings provide a conceptual basis for future development of therapeutic strategies that co-target EA and VM, with prior work demonstrating the feasibility of EGFR-directed agents that affect both processes [13,33];). Here, we provide the in vitro/in vivo arm, which serves as a focused proof of concept, while the DepMap analysis extends across the broader TNBC landscape.

## 2. Materials and Methods

### 2.1. Cell Lines

TNBC cell lines, namely, MDA-MB-231 human adenocarcinoma cells, MDA-MB-231-4175 human lung-tropic cells, and MDA-MB-468 human adenocarcinoma cells, were used in the in vitro and in vivo experiment models ([33]). Non-TNBC (control) 3B-11 mouse lymphoid endothelial cells were a kind gift from the Albert Einstein school of medicine. Cell authentication was performed by the suppliers. The 3B-11 cells were cultured in Dulbecco’s modified eagle medium (DMEM, 4.5 g/L of high glucose, Diagnovum, Greifswald, Germany, catalog no. D009), supplemented with 10% FBS (Diagnovum, Germany, catalog no. D151), 100 U/mL of L-glutamine (Biowest, Nuaillé, France, catalog no. MS027K2006), and 100 U/mL of penicillin–streptomycin (100×) (Diagnovum, Germany, catalog no. D910). MDA-MB-231, MDA-MB-231-4175, and MDA-MB-468 cells (all TNBCs) were cultured in RPMI-1640 medium (Diagnovum, Germany, catalog no. D039), supplemented with 10% FBS, 100 U/mL of L-glutamine, and 100 U/mL of penicillin–streptomycin. All cells were grown in incubators at 37 °C and 5% CO_2_.

### 2.2. VM In Vitro Tube Formation Assay

For each of the cell lines, the tube formation assay was performed inside pre-chilled 48-well cell culture microplates (Corning Falcon, Corning, NY, USA, catalog no. 353078). The pre-chilled 48-well microplates were coated with 100 µL of unpolymerized Matrigel (Corning, NY, USA, catalog no. 3562237) and incubated at 37 °C for 1 h with 5% CO_2_. Each of the cell lines was harvested via trypsinization, and approximately 7.5 × 10^5^ cells were resuspended in 450 µL of the respective media and seeded onto the matrigel. Tube formation for each of the cell lines was examined using the IncuCyte (Sartorius, Göttingen, Germany, SX5) live-cell analysis system, and 10×-magnified images were captured every 30 min for 2.5 h.

### 2.3. ImageJ Analysis

The Angiogenesis Analyzer plug-in was integrated into ImageJ (version 1.49v), a Java-based image-processing program developed at the National Institutes of Health (NIH) [34], to quantify nodes, meshes, and branches in the VM tube formation assay, as previously described by [33]. Nodes (pixels with ≥3 neighbors) and meshes (closed polygonal areas) were used as the primary readouts of VM competence; branches (lines with free extremities) were reported as a secondary descriptor of cell morphology.

### 2.4. Single-Cell Motility Tracking

For each of the cell lines, 10 randomly selected cells were tracked as they migrated across the matrigel using the Fiji (ImageJ, version 1.54f) image-processing package [35], with two technical replicates per biological experiment and five independent biological experiments, yielding 100 single-cell tracks per cell line in total. The per-replicate means were used for statistical analysis (*N* = 5 biological replicates). The migration data points were extrapolated to generate trajectory plots and to quantify velocity, accumulated distance, and Euclidean distance using the Chemotaxis and Migration Tool (ibidi, version 4.3.2) [33]. The accumulated distance corresponds to the total length of the curved path across which the cell has migrated, and the Euclidean distance corresponds to the linear distance the cell has migrated. Path tortuosity was calculated as the ratio of accumulated distance to Euclidian distance. A path tortuosity with a ratio of 1 was considered straight paths, and ratios higher than 1 were considered more convoluted paths.

### 2.5. Statistical Analysis

All quantification data are presented as mean ± SEM (Standard Error of the Mean) derived from five independent biological experiments. All comparisons for in vitro VM tube formation assay and the cell migration metrics (velocity, accumulated distance, Euclidean distance, and path tortuosity) between the control and test groups were conducted using one-way ANOVA with Dunnett’s post hoc test. All statistical analyses and graphical representations were performed and created, respectively, using GraphPad Prism (Version 5.0, GraphPad Software Inc., San Diego, CA, USA). For the DepMap-based transcriptomic analyses, signature relationships were assessed via Pearson correlation; TF regulon enrichment was assessed via one-sided Fisher’s exact tests with Benjamini–Hochberg FDR correction, and regulon-activity subgroup comparisons were made using one-sided Mann–Whitney U tests; and hypoxia–VM signature relationships in TCGA-BRCA and METABRIC were assessed via Spearman correlation. Statistical significance was set at *p* < 0.05, with ns = not significant, * denoting *p* ≤ 0.05, ** denoting *p* ≤ 0.01, *** denoting *p* ≤ 0.001, and **** denoting *p* ≤ 0.0001.

### 2.6. Orthotropic TNBC Xenograft Model

An in vivo orthotopic TNBC xenograft model was established by injecting human MDA-MB-231 and MDA-MB-4175 cells into the mammary fat pads of NOD scid gamma (NSG) immunodeficient mice (*N* = 5). Tumor growth was monitored for 30 days, starting with 2 days post-implantation. Then, mice were euthanized, and the primary tumors were harvested and mounted in optimal-cutting-temperature (OCT) blocks (Tissue-TEK OCT Compound, catalog.no. 4583).

### 2.7. Immunofluorescence Staining of In Vivo Tumor Sections

OCT-embedded xenograft tumors were snap-frozen in liquid nitrogen and sectioned using cryostat. The sections were carefully mounted onto slides for fixation and permeabilization with ice-cold methanol for 10 min at room temperature (RT). The slides were then washed three times with 1× PBS (Sigma Aldrich, St. Louis, MO, USA, Catalog no. P7059-IL) and blocked with freshly prepared 5% bovine serum albumin (BSA in 1× PBS, Sigma Aldrich, USA Catalog no. A7906-50G) for 1 h. A cocktail of primary antibodies against CD31 (Biotin Rat anti-mouse CD31 clone MEC 13.3, BD Pharmigen, San Diego, CA, USA, Catalog no. 553371) and hLaminin5 (monoclonal mouse anti-Laminin clone LAM-89, Sigma Aldrich, USA, Catalog no. L8271) was applied to the slides and incubated overnight at RT. Following incubation, a cocktail of secondary antibodies (Streptavidin Alexa Fluor 594, Invitrogen, Carlsbad, CA, USA, Catalog no. S32356; Goat anti-mouse Alexa Fluor 488, Invitrogen, USA, Catalog no. A11029) was added to the sections, and they were incubated for 2 h at RT. The sections were then washed with 1× PBS and mounted with Fluoro-Gel II containing DAPI (Electron Microscopy Sciences, Hatfield, PA, USA catalog no. 17985-51). The slides were imaged using 10× and 40× objectives via an Olympus Laser Confocal Microscope, Evident, Waltham, MA, USA.

### 2.8. Ethical Compliance

All animal experiments were conducted under ethical compliance with the guidelines approved for the Institutional Animal Care and Use by the ethics committee at the University of Miami Miller School of Medicine (Miami, FL, USA), permit # 21-155 (21 July 2021).

### 2.9. Text-Mining and Cancer Dependency Map DepMap Portal

Gene signatures associated with VM, EA, and underlying contributing sub-processes, including EMT, CSC, ECM remodeling, and vessel stability (VS), were identified using the Geneshot web tool [36]. Gene sets derived from these signatures were then used to extract baseline gene expression data of breast cancer cells and endothelial cells (ABMT0725, the human endothelial line available in DepMap) from the DepMap portal (https://depmap.org/portal/ (3 December 2025)). From each set, the top 20 genes were identified based on their reported roles in the literature, together with evidence of enriched expression in DepMap cell line metadata (Supplementary Table S1). The curated list of genes was subsequently used for downstream analyses of different mechanistic modules and graphical visualization using R script (R studio, version R 4.5.2).

### 2.10. In Silico Transcription-Factor (TF) Regulon Prioritization

To nominate candidate transcriptional regulators of the VM-competent state for future functional validation, eight EMT- and stem-cell-associated transcription factors implicated in VM biology (TWIST1, ZEB1, ZEB2, SNAI1, SNAI2, FOSL1, SOX2, and FOXC2) were assembled with research-curated direct target sets, restricted to the VM-associated gene panel (Section 2.9). Regulon enrichment among genes up-regulated in VM-competent versus VM-incompetent lines was assessed across the DepMap TNBC panel and, separately, for the three anchor lines (ABMT0725, MDA-MB-231, and MDA-MB-468). Per-line regulon activity was computed as the mean of gene-wise z-scored target expression across the 71 breast and endothelial cell lines profiled.

### 2.11. Hypoxia–VM Signature Correlation in Patient Cohorts and Hypoxia-Induced VM-Gene Response in TNBC Cell Lines

Expression data and intrinsic molecular subtype annotations for TCGA-BRCA (Pan-Cancer Atlas 2018) and METABRIC were retrieved from cBioPortal [37]. Per-sample hypoxia and VM activity scores were computed as the mean of gene-wise z-scored expression across the MSigDB HALLMARK_HYPOXIA gene set [38] and a curated VM-program panel (Supplementary Figure S3 legend), and hypoxia–VM correlations were assessed within each subtype. Hypoxia-induced expression changes in MDA-MB-231, MDA-MB-468, and BT-549 cells were derived from the GSE111653 Salmon-quantified count matrix [39] as the log_2_ fold-change between hypoxic and normoxic conditions, with VEGFA and CA9 included as HIF-axis positive controls.

## 3. Results

### 3.1. VM Competence in TNBC Is Heterogeneous and Coupled to Endothelial-like Directed Migration

This study evaluates the VM competence of human TNBC cells with respect to forming tube networks and compares this ability with control EA. For this purpose, 3 types of TNBC cells and 1 type of endothelial (control) cell were used (Table 1).

**Figure 1:**
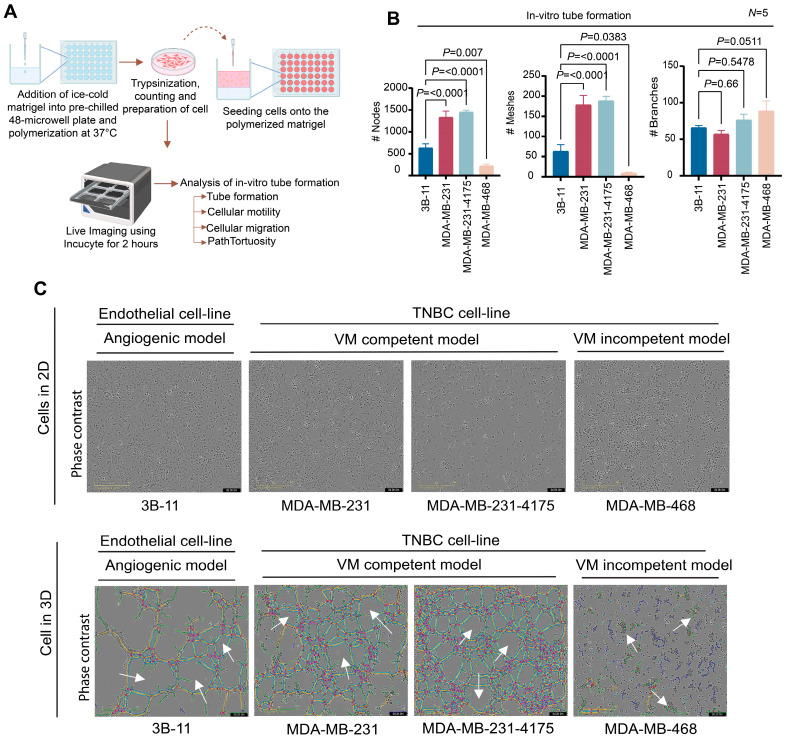
TNBC cells displaying heterogeneous VM competence in a 3D tube-formation assay. (**A**) Schematic of the workflow of the in vitro 3D tube-formation assay. (**B**) Quantification of nodes, meshes, and branches of 3B-11 (control) and MDA-MB-231/MDA-MB-231-4175/MDA-MB-468 TNBC cells (experiment) during and after tube formation (data are presented as means of 5 independent experiments). (#) denotes the count in number. (**C**) Representative phase-contrast micrographs of both angiogenic and TNBC lines in 2D and 3D under 10× magnification. White arrows indicate the mesh-like network, formed by VM. Black bars correspond to time stamps (days (d), hours (h), minutes (m)), and scale bars correspond to 400 µm. Statistical significance was assessed using one-way ANOVA with Dunnett’s post hoc multiple-comparisons test against the 3B-11 control. Data are presented as means ± SEMs, with *p* < 0.05 (*p* > 0.05—ns (not significant), *p* ≤ 0.05—*, *p* ≤ 0.01—**, and *p* ≤ 0.001 or 0.0001—*** or ****).

All cell lines were seeded onto 3D Matrigel, a basement membrane-like matrix, and monitored for tube-network formation (Figure 1A). Following seeding, 3B-11 endothelial cells rapidly migrated and formed tubular networks within 2 h (Supplementary Video S1). By 3 h, part of this network remained detectable, although many structures had partially dwindled, potentially reflecting cell death or reduced structural stability. Based on these observations, a time point of 2 h was selected as the standard endpoint for subsequent comparisons of VM competence across all cell lines.

Notably, two of the TNBC cells, MDA-MB-231 and MDA-MB-231-4175, formed extensive vessel-like networks and were VM-competent. In fact, these cells displayed a high number of nodes and meshes and a similar number of branches relative to the endothelial control (Figure 1B). In contrast, MDA-MB-468 showed numerous extended short segments that failed to converge into junctions or closed loops, yielding a high branch count alongside markedly lower quantities of nodes and meshes, a pattern indicative of a fragmented, non-connecting morphology rather than productive VM network formation when compared to both the 3B-11 control and the other TNBC cell lines, and it was therefore classified as a VM-incompetent TNBC model (Figure 1B). However, MDA-MB-468 displayed the highest number of branch points among the TNBC cell lines, indicating that not all network-related parameters correlate equally with overall VM competence. The VM heterogeneity across the three TNBC lines examined suggests that VM competence is not inherent and may be supported by a distinct underlying transcriptional program.

Our in vitro tube formation assay further revealed that the MDA-MB-231 and MDA-MB-231-4175 cells underwent guided migration across the matrigel to assemble VM-like channels, whereas this behavior was largely absent in the MDA-MB-468 cells (Figure 1C, Supplementary Videos S1–S4). Based on this observation, we hypothesized that VM-competent TNBC cells co-opt an endothelial-like mechanosensitive migration program that enables directed movement through the extracellular matrix and promotes VM channel formation.

**Figure 2:**
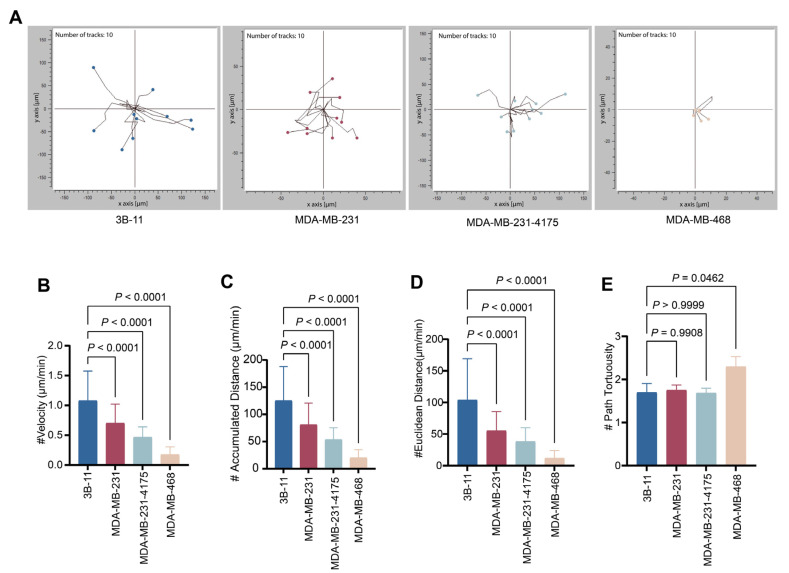
VM-competent TNBC cells recapitulate endothelial-like directed migration, while VM-incompetent cells display slow, meandering motility. (**A**) Trajectory plots representing single-cell motility tracks of 3B-11, MDA-MB-231, MDA-MB-231-4175, and MDA-MB-468 (10 representative tracks are shown per cell line for illustration; full quantitative analysis in B–E is based on *N* = 5 biological replicates, with 100 tracks per cell line). Quantification graphs showing overall (**B**) velocity, (**C**) accumulated distance, (**D**) Euclidean distance, and (**E**) path tortuosity for 3B-11 versus each of the TNBC cells, namely, MDA-MB-231, MDA-MB-231-4175, and MDA-MB-468 (*N* = 5). (#) represents count in number. Statistical significance was assessed via one-way ANOVA with Dunnett’s post hoc multiple-comparisons test against the 3B-11 control (data are presented as means ± SEM—*, *p* < 0.05; **, *p* < 0.01; and ***, *p* < 0.001).

To test this, we performed single-cell motility-tracking analysis (Figure 2A). This analysis showed that the 3B-11, MDA-MB-231, and MDA-MB-231-4175 cells exhibited outward-directed trajectories, in contrast to the MDA-MB-468 cells, which did not exhibit this organized migratory pattern. Consistent with this behavior, additional migration metrics, including velocity, accumulated distance, and linear distance, demonstrated that the patterns of the MDA-MB-231 and MDA-MB-231-4175 cells closely resembled the migratory behavior of endothelial 3B-11 cells, whereas this was not the case for the MDA-MB-468 cells (Figure 2B–D).

The poor mobility of the MDA-MB-468 cells was also consistent with their limited ability to form VM network structures, suggesting that efficient spatial exploration of the 3D matrix is required for VM channel assembly. Notably, the 3B-11, MDA-MB-231, and MDA-MB-231-4175 cells displayed similarly low path tortuosity values (approximately 1.5 and closer to 1), indicative of guided or directed migration. In contrast, the MDA-MB-468 cells showed higher path tortuosity values, consistent with a more meandering and less efficient migratory pattern (Figure 2E).

### 3.2. Orthotopic TNBC Xenografts Recapitulate VM In Vivo, with Laminin-5^+^ and CD31^+^ Vessels Coexisting as Spatially Distinct and Mosaic Structures

**Figure 3:**
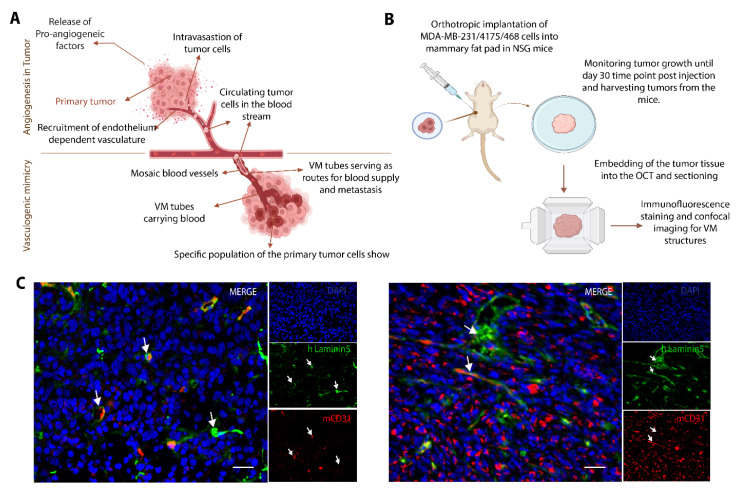
VM and EA are spatially distinct yet co-exist within the same TNBC tumor in vivo. (**A**) Schematic representation of tumor vascularization, first through endothelial-dependent angiogenesis and later through VM. Both types of vascularization may co-exist. (**B**) Experimental workflow of our in vivo orthotropic implantation of human TNBC cells (MDA-MB-231/MDA-MB-4175) into the mammary fat pads of NSG immunodeficient mice, tumor harvesting, OCT embedding, and immunohistochemical staining. (**C**) Representative immunohistochemical micrograph of tumor sections stained with DAPI nuclear stain (in blue), CD31^+^ endothelial marker (in red), and Laminin-5 VM marker (in green) under 40× objective magnification. White arrows indicate mosaic vessel-like structures, and the white bar is a scale bar (20 µm).

VM was assessed in vivo using orthotopic TNBC xenografts derived from MDA-MB-231 and MDA-MB-231-4175, the same VM-competent cell lines that formed functional tube networks in vitro (Figure 1), to test whether their functional VM capacity translated into histological hallmarks of VM in tissue, as shown in the schematic workflow in Figure 3B. Notably, the overall architecture of the vessel-like conduits formed by the VM-competent TNBC cells in vitro resembled that of classical EA networks (Figure 1 and Figure 3A); this morphological similarity is restricted to channel geometry and does not imply shared cellular origin or regulatory mechanisms, since the channels are constructed entirely by tumor cells rather than by endothelial cells. Interestingly, the in vivo tumor models exhibited a mixture of blood vessels and mosaic vessels, with some harboring CD31^+^, a marker of classical EA, and with others expressing Laminin-5^+^/CD31^−^, a marker of VM-like channels unrelated to EA (Figure 3C). Notably, some of the vessels were labeled by both markers, CD31^+^ and Laminin-5^+^, and suggest that these structures can co-exist as mosaic vessels.

### 3.3. VM and EA Share a Partial Transcriptional Core but Diverge Toward Distinct Lineage-Specific Programs

**Figure 4:**
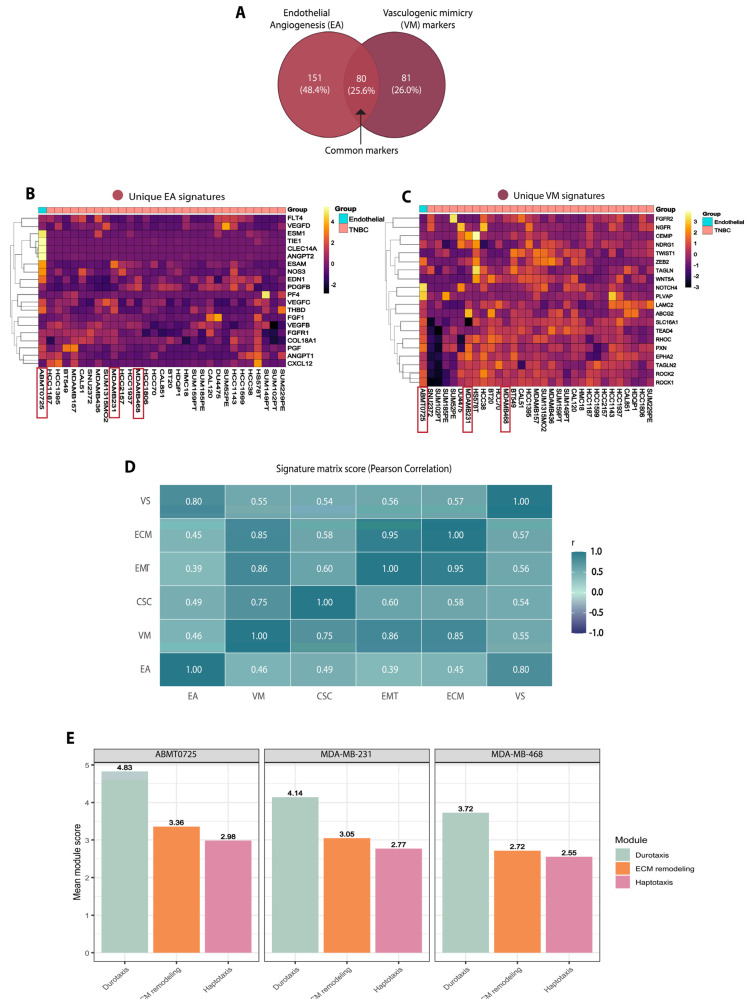
DepMap-based transcriptomics reveals that VM and EA share a partial gene overlap yet diverge toward distinct downstream programs in breast cancer. (**A**) Venn diagram highlighting distinct and common genes in vascularization processes of EA and VM programs. (**B**,**C**) Heatmaps showing basal enrichment of 20 curated genes linked with EA and VM among endothelial and TNBC lineages. (**D**) Pearson correlation matrix of EA and VM genes linked with cancer stem cells (CSCs), epithelial–mesenchymal transition (EMT), extracellular matrix remodeling (ECM/mesenchymal), and vessel stability (VS). (**E**) Graph scoring association with Durotaxis, ECM remodeling, and Haptotaxis modules (transcriptional enrichment of durotaxis, ECM remodeling, and haptotaxis-related genes from DepMap expression data, not direct mechanical measurements) among ABMT0725, MDA-MB-231, and MDA-MB-468 cell lines based on DepMap metadata.

Next, we sought to define the transcriptional and mechanistic relationship between these two parallel tumor vascularization strategies and identify genes associated with VM and classical EA. Text-mining-based curation of EA and VM gene sets revealed a partial overlap of 80 genes associated with EA (231 genes) and VM (161 genes) (Figure 4A). For the DepMap analysis, we used the top 20 unique genes associated with EA ABMT0725, a human endothelial cell line, as a control model for angiogenesis instead of 3B-11, the immortalized mouse cell line used in our in vitro experiments. Consistent with lineage-specific enrichment, the top 20 genes uniquely associated with the EA and VM programs were also enriched in ABMT0725 and in the TNBC cluster, respectively, suggesting a higher degree of codependence than previously thought (Figure 4B,C).

To test if VM and EA represent distinct alternative vascularization strategies, we analyzed their correlation with other tumor-associated programs, such as vessel stability (VS), cellular stemness (CS), epithelial-to-mesenchymal transition (EMT), and extracellular matrix (ECM) remodeling (Figure 4D). The top 20 gene enrichment expressions strongly associated with each of the tumor-associated programs from the DepMap dataset were considered for assessing correlation. Notably, in the Pearson signature correlation matrix (Figure 4D), EA was most correlated with VS (0.55), while VM, on the other hand, showed higher correlation with CSC (0.75), EMT (0.86), and ECM remodeling (0.85). These results indicate that VM is associated with a stem-like, mesenchymal, and matrix-remodeling tumor state that is transcriptionally related to, but markedly distinct from, classical EA.

To nominate candidate transcriptional regulators of the VM state, we additionally performed an in silico TF regulon enrichment analysis across 22 TNBC cell lines (Supplementary Figure S2). Five EMT and stem-cell-associated transcription factors—SNAI2, SNAI1, FOXC2, FOSL1, and ZEB1—showed significant enrichment of their target regulons in the VM-competent transcriptional state (FDR < 0.05, one-sided Fisher’s exact test with Benjamini–Hochberg correction), leading to their nomination as candidate regulators for future functional validation. These analyses resulted in the nomination of candidate regulators of the VM transcriptional state and provide a prioritized shortlist for future mechanistic interrogation; causal contribution of individual factors will require targeted perturbation studies with VM-specific phenotypic readouts. To test whether the VM transcriptional program is responsive to a known physiological driver, we examined the relationship between hypoxia and VM signature activity in two independent patient cohorts and in a public TNBC hypoxia-perturbation dataset (Supplementary Figure S3). The hypoxia and VM signatures were significantly positively correlated within the basal subgroup in both TCGA-BRCA (Spearman ρ = +0.33, *p* = 1 × 10^−5^, *n* = 171) and METABRIC (ρ = +0.28, *p* = 5 × 10^−5^, *n* = 209), and VM-program genes (*LAMC2*, *MMP2*, *MMP9*, *ZEB1*, *TWIST1*, *VIM*, *SNAI1*, and *EPAS1*) showed hypoxia-induced upregulation under 1% O_2_ across MDA-MB-231, MDA-MB-468, and BT-549 (GSE111653), with MDA-MB-468 displaying the strongest induction and robust *CA9* induction across all three lines confirming intact HIF-axis function.

As such, the genes associated with these processes could serve as potential targets in drug design. In agreement with these findings, ABMT0725, the endothelial cell line used for transcriptomic comparison, and MDA-MB-231 cells both showed elevated mean transcriptional signature scores for durotaxis- and mechanotransduction-related genes relative to ECM remodeling and haptotaxis, consistent with enrichment of a mechanosensitive migratory transcriptional module (Figure 4E). By contrast, MDA-MB-468 cells scored poorly across all three modules, particularly for durotaxis, consistent with their reduced motility and VM-incompetent phenotype.

Collectively, these data suggest that acquisition of endothelial-like migratory behavior is a key feature of VM-competent TNBC cells, enabling them to efficiently traverse the 3D matrix and organize into VM-like channels.

### 3.4. VM Competence Is a TNBC-Enriched Transcriptional State: t-SNE Clustering Reveals Two Distinct VM Clusters with Subtype-Dependent Gene Enrichment

**Figure 5:**
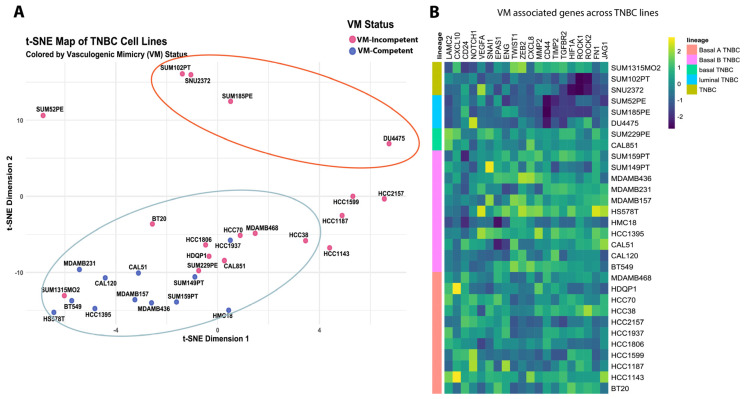
VM competence in TNBC is transcriptionally encoded: t-SNE clustering and gene expression profiling define two distinct VM states. (**A**) t-SNE map of human breast cancer cell lines in transcriptional space, showing clustering of TNBC lines based on VM status. (**B**) Heatmap displaying VM-associated gene enrichment across TNBC clusters.

To investigate if VM is associated with TNBC, breast cancer cell lines from DepMap were clustered using VM-associated gene signatures, lineage, and subtype annotations. Notably, TNBC and non-TNBC cell lines were segregated into two main, partially overlapping clusters, consistent with a continuum of VM-related transcriptional states. Nevertheless, most TNBC cell lines showed stronger association with VM competence than non-TNBC cell lines (Figure 5A and Figure S1A).

Importantly, MDA-MB-231, but not MDA-MB-468, mapped within the VM-competent region of this transcriptomic space, in agreement with the tube formation assay, supporting our clustering. Baseline expression analysis of a curated 20-gene VM signature, selected based on established roles in VM and consistent enrichment across the dataset, revealed higher overall VM-associated gene expression in TNBC clusters than in non-TNBC clusters (Figure 5B and Figure S1). A minority of non-TNBC lines, such as HCC1569, HCC1954, HMEL, and JIMT1, also displayed modest enrichment of VM-associated genes. Collectively, these findings show that endothelial cells and VM-competent TNBC lines form robust tube-like networks in 3D cultures, whereas VM-incompetent TNBC cells do not, and that this phenotypic VM competence corresponds to global transcriptional enrichment of VM-associated genes prevalent in most TNBCs.

## 4. Discussion

In this study, we combined a foundational in vitro tube formation assay with orthotopic xenograft immunofluorescence and DepMap-based transcriptomic analyses to characterize the heterogeneity of vasculogenic mimicry across three molecularly distinct TNBC cell lines and examine its relationship with classical EA. Below, we discuss how these complementary observations refine the conceptual distinction between VM and EA and what they imply for TNBC vascular biology. As a control for classical angiogenesis, we used the immortalized endothelial cell line 3B-11, which produced canonical endothelial vessel networks. Furthermore, immunohistochemical analysis of TNBC xenografts revealed that EA and VM networks can be distinguished by CD31 and laminin-5 markers, respectively. Interestingly, these markers occasionally co-localized within mosaic-like vessels, suggesting coexistence of endothelial and tumor-cell-derived vascular structures. Finally, using DepMap-based transcriptomic analysis, we examined signature overlaps among CSC, EMT, ECM remodeling, and mechanosensitive migration programs involved in channel formation, revealing only partial overlap between EA- and VM-associated transcriptional signatures.

VM is a tumor-cell-driven vascularization strategy that substitutes for classical endothelium-dependent angiogenesis. It was first noted in uveal melanoma [2,40], and thereafter it was found in multiple other aggressive cancers, including triple-negative breast cancer (TNBC) [41,42,43]. VM-associated channels have been implicated in tumor invasion, metastasis, and therapeutic resistance. VM has been underscored as one of the most concerning causes of disease relapse and declining survival outcomes among TNBC patients [44,45,46]. Although VM can coexist with classical angiogenesis within the same tumor, the two processes remain biologically distinct. While both endothelial- and tumor-cell-driven vascularization ultimately support tumor perfusion, our integrated in vitro tube formation assays, orthotopic xenograft models, and transcriptomic analyses demonstrate that TNBC cells use different, tumor-intrinsic mechanisms to establish vessel-like networks. Critically, the two processes differ not only in terms of molecular signatures but also in terms of the cell of origin and regulatory logic: EA is a host-driven response in which endothelial cells, recruited by tumor-secreted angiogenic factors (e.g., VEGF), assemble CD31^+^/CD34^+^ vessels, whereas VM is a tumor-intrinsic process in which malignant cells themselves adopt endothelial-like plasticity and deposit Laminin-5^+^ basement membrane to form endothelium-independent channels. Architectural similarity between the two structures should therefore not be interpreted as mechanistic equivalence.

VM is strongly associated with aggressive tumors, yet several studies indicate that it is not a universal phenotype. Instead, VM shows substantial interpatient and intratumoral heterogeneity, even within the same cancer type [9,10,11]. Consistent with this, our in vitro tube formation assay in a 3D matrigel matrix (Figure 1B,C) showed that although MDA-MB-231, MDA-MB-231-4175, and MDA-MB-468 all belong to the TNBC subtype, they differ markedly in their ability to form VM structures. This finding reinforces the idea that VM requires acquisition of a specialized VM-competent state and is therefore restricted to discrete tumor cell subpopulations and does not represent a uniform property of all TNBC cells. Prior studies have established causal roles for EMT transcription factors and the MMP/laminin-5γ2 axis in VM across multiple tumor contexts, including TWIST1/ZEB1-driven EMT in TNBC [47] and MMP-2/MT1-MMP-mediated cleavage of laminin-5γ2 in melanoma [48,49], providing mechanistic context for the candidate regulators nominated by our analysis. Our in vivo data further showed that TNBC xenografts contain, among others, complex mosaic vascular networks in which classical CD31^+^ endothelial vessels coexist with Laminin-5^+^/CD31^−^ VM-like channels. This finding is consistent with previous reports showing that VM and endothelium-dependent vessels can function in parallel within aggressive tumors [50,51]. Importantly, the apparent co-localization of Laminin-5^+^ and CD31^+^ markers within individual vessel-like structures is consistent with, though does not by itself demonstrate, anatomical continuity between tumor-derived and endothelial segments. If confirmed by future quantitative spatial and perfusion-based analyses, such continuity would suggest that VM channels are integrated with the host endothelial vasculature and contribute to tumor perfusion. By integrating text-mining-based gene curation with lineage-resolved DepMap analysis, we found that VM is supported by a transcriptional program that only partially overlaps with classical EA. This is in line with the concept that tumor cells that acquire epithelial-to-endothelial-like plasticity selectively adopt certain endothelial traits while remaining molecularly distinct from true endothelial cells [23,52]. Notably, VM in TNBC was more strongly associated with cancer-stem-cell, EMT-, and ECM-remodeling signatures than with EA itself, suggesting that these programs are associated with VM formation and metastatic competence, although mechanistic validation through functional perturbation studies will be required to confirm whether there is a causal contribution. Consistent with this association, based on our TF regulon analysis (Supplementary Figure S2), we nominate SNAI1, SNAI2, FOXC2, FOSL1, and ZEB1 as candidate transcriptional regulators of the VM state, and our hypoxia analysis (Supplementary Figure S3) shows that the VM program is co-regulated with hypoxia in patient tumors and inducible under low-O_2_ conditions in TNBC cell lines.

The heterogeneity observed was also reflected in our TNBC t-SNE map, where cell lines were stratified based on lineage and the expression profiles of curated VM-associated genes. VM competence was enriched among TNBC lines relative to their non-TNBC counterparts, although substantial variability was evident within the TNBC cohort itself. Consistent with our 3D functional assays, only a subset of TNBC cell lines demonstrated VM-forming ability, with MDA-MB-231 exhibiting pronounced VM activity compared to the largely non-competent MDA-MB-468 line. Notably, VM-competent and VM-incompetent lines formed contiguous clusters rather than dispersed outliers, indicating the presence of a coherent VM-associated transcriptional program. These findings suggest that VM-related gene expression patterns may serve as the basis for predictive-scoring approaches capable of identifying aggressive breast cancer models that have not yet been experimentally characterized [53]. The distinction between VM-competent and VM-incompetent states was not absolute, suggesting that VM capacity represents a dynamic and potentially reversible trait that may be acquired or lost during tumor evolution or in response to therapeutic and microenvironmental influences. In line with this concept, VM-competent TNBC lines exhibited broad enrichment of the VM-associated transcriptomic signature, whereas this enrichment was markedly diminished in the VM-incompetent counterparts. These observations indicate a strong, though not exclusive, association between the VM-related transcriptional program and the VM-competent state [50]. Several limitations should be acknowledged. First, the functional characterization is based on three TNBC cell lines and represents a proof of concept; these lines were chosen as functional anchors covering both ends of the VM-competence spectrum. To extend our work beyond this three-line comparison, we performed transcriptomic analyses across 22 TNBC and 71 breast cancer cell lines from DepMap (Supplementary Figure S2), correlated gene VM signature with hypoxia across tumor datasets from TCGA-BRCA and METABRIC tumors (Supplementary Figure S3A,B), and examined hypoxia-induced VM-gene induction in MDA-MB-231, MDA-MB-468, and BT-549 (Supplementary Figure S3C). Future wet-lab validation in additional TNBC lines and patient-derived xenografts will further consolidate these observations. Second, our xenografts lack a competent immune system and do not capture immune–tumor influences on vascular remodeling. Third, the transcriptomic associations with CSC, EMT, ECM remodeling, and mechanosensitive migration programs remain correlative, and genetic perturbation is required in order to establish causality. Our analyses establish correlative and regulon-level associations but do not directly test causality; targeted perturbation of nominated regulators with VM-specific phenotypic readouts (tube formation and channel assembly) will be required to confirm functional contribution to VM in TNBC. Fourth, the translational relevance of VM-associated signatures awaits testing in patient-derived models and clinical settings. Although the present study includes complementary in silico TF regulon (Supplementary Figure S2) and hypoxia-perturbation (Supplementary Figure S3) analyses that nominate candidate regulators and demonstrate co-regulation of the VM program with hypoxia, these analyses remain correlative; experimental perturbation studies including TF loss- and gain-of-function and hypoxia-dependent VM modulation will be required to establish causality. Mechanosensitivity in VM-competent cells was inferred from transcriptomic enrichment of durotaxis- and haptotaxis-related programs and from migration patterns on Matrigel, but it was not directly tested; migration assays on substrates of defined stiffness, live readouts of mechanotransduction (e.g., nuclear YAP/TAZ localization), and perturbation of mechanosensitive pathways will be required to establish a causal role for mechanosensitive guidance in VM channel formation.

Finally, functional VM competence was assessed primarily via Matrigel tube formation and complemented by in vivo xenograft immunofluorescence. While the detection of Laminin-5^+^/CD31^−^ channels and Laminin-5^+^/CD31^+^ mosaic vessels in xenograft tissue is consistent with VM channels being integrated into the perfused tumor vasculature, direct measurements of perfusion, lumen patency, and intravascular flow were not performed. Confirmation via intravital imaging, fluorescent tracer perfusion, or contrast-enhanced vivo imaging will be important for definitively establishing the functional vascular behavior of VM channels in future work. Additionally, in our in vitro functional assays, we used the murine lymphoid endothelial line 3B-11, while in the DepMap transcriptomic comparison, we used the human endothelial line ABMT0725, a practical constraint reflecting line availability, not experimental design; future work must incorporate primary human vascular endothelial cells (e.g., HUVEC and HMVEC) for both functional and transcriptomic benchmarks. Nevertheless, these findings support a model in which TNBC exploits two parallel but mechanistically distinct vascularization strategies: VM rooted in a tumor-intrinsic, endothelial-like plastic state, and EA, optimized for tumor maintenance through host vascular support. This dual vascular strategy may help explain why anti-angiogenic therapies targeting endothelial VEGF signaling often fail to suppress, and may even indirectly favor, VM-rich TNBC tumors. Our results therefore argue for therapeutic strategies that simultaneously target EA and the tumor-intrinsic circuits that support VM, particularly CSC-, EMT-, and ECM-remodeling pathways [13,33,54,55].

An additional key finding of our study is that migration patterns and transcriptomic features consistent with endothelial-like mechanosensitivity distinguish VM-competent from VM-incompetent TNBC cells. Single-cell tracking revealed that VM-competent MDA-MB-231 and MDA-MB-231-4175 cells recapitulate several hallmarks of endothelial migration, including high velocity, greater displacement, low path tortuosity, and outward-directed trajectories that resemble those of 3B-11 endothelial cells. In contrast, VM-incompetent MDA-MB-468 cells displayed slower, highly meandering migration and failed to assemble robust VM networks. Previous work has shown that VM-positive tumors are highly responsive to ECM composition and stiffness, enabling guidance through haptotaxis and durotaxis [56]. In agreement with this, our signature scoring analysis showed that both ABMT0725 and MDA-MB-231 were more strongly enriched with respect to durotaxis-related features than ECM remodeling or haptotaxis modules. Together, these findings are consistent with a model in which VM-competent TNBC cells engage an endothelial-like mechanosensitive migration program that enables guidance-based exploration of stiffness and adhesion gradients within the 3D matrix, thereby promoting organization into VM-like channels—although direct experimental validation on substrates of defined stiffness and through perturbation of mechanotransduction pathways will be required to confirm this inference. Importantly, this co-option of endothelial-like migratory features does not imply that VM-competent cells acquire true endothelial identity; rather, it reflects selective adoption of specific endothelial traits within a tumor-cell-intrinsic regulatory framework.

Taken together, our findings, while based on a functionally anchored panel of three TNBC lines complemented by transcriptomic analyses spanning several breast cancer cell lines and primary tumors on a limited panel of TNBC lines, support the view that VM constitutes a distinct, heterogeneous, and therapy-relevant state in TNBC rather than a uniform property of the subtype and provide a framework for future studies across larger TNBC cohorts. We propose that rare, highly plastic tumor cells progressively acquire VM competence through coordinated stem-like, mesenchymal, and mechanosensitive features, allowing them to expand, potentially under hypoxic or therapeutic pressure. This VM-competent state supports the formation of mosaic vascular networks in which tumor-derived channels function alongside classical endothelial vessels, potentially contributing to sustained perfusion, resistance to anti-angiogenic therapy, and metastatic progression. Although VM shares some transcriptional features with EA, it is anchored in distinct tumor cell programs involving CSC, EMT, and ECM remodeling. These features may provide a basis for prospective VM scoring intended to identify high-risk TNBC states.

Several key questions remain unexplored: What factors drive VM competence switching in specific TNBC cells within a heterogenous tumor? Do VM-competent subclones exhibit distinct metabolic rewiring to sustain channel perfusion? How does the immune microenvironment differ between VM and endothelial vessels? Does VM signature intensity predict response to chemotherapy or immunotherapy in TNBC patients? A dedicated spatial–vascular study combining quantitative regional mapping of CD31^+^, Laminin-5^+^, and mosaic vessel densities with 3D continuity and perfusion analyses in immune-competent or humanized models will be valuable for defining intra-tumor vascular heterogeneity in TNBC. We can investigate these open key questions by integrating breast cancer gene expression data, coupled with VM metabolism and immune analysis with VM scores, to reveal the vitality of immune-privileged niches in VM. Thus, developing an integrated multivariate VM risk model will in turn aid in the design of anti-VM drugs that can be integrated in the TNBC cancer treatment regime and guide precision therapy in relapsed TNBC cohorts.

## 5. Conclusions

Our results indicate that vasculogenic mimicry competence is heterogeneous across the three TNBC cell lines examined, with MDA-MB-231 and MDA-MB-231-4175 forming robust vessel-like networks in 3D Matrigel, whereas MDA-MB-468 does not. DepMap-based transcriptomic analysis showed that VM and endothelial angiogenesis share only a partial gene-level overlap, with VM more strongly associated with cancer stem cell, epithelial-to-mesenchymal transition, and ECM remodeling signatures, and EA more closely associated with vessel stability. VM-competent TNBC cells also displayed migration patterns and transcriptomic features consistent with endothelial-like mechanosensitivity. Together, these findings suggest that VM represents a distinct, heterogeneous transcriptional state in TNBC that complements classical angiogenesis and may provide a conceptual basis for future studies on dual-targeting strategies in this disease.

## Figures and Tables

**Table 1:** Cell types used in this study.

Name	Cells	Source
3B-11 (control)	Immortalized lymphoid endothelia	Mouse
MDA-MB-231 ^†^	TNBC * with mesenchymal phenotype	Human
MDA-MB-231-4175 ^†^	TNBC * with mesenchymal phenotype, derived from lung-tropic MDA-MB-231 ^†^	
MDA-MB-468 ^†^	TNBC * with epithelial phenotype	

* Triple-negative breast cancer. ^†^ Acronym of M.D. Anderson—Metastatic Breast.

## Data Availability

The original contributions presented in this study are included in the article/Supplementary Materials. Further inquiries can be directed to the corresponding authors.

## Supplementary Materials

The following supporting information can be downloaded at https://www.mdpi.com/article/10.3390/cancers18111789/s1. Figure S1. Transcriptional clustering and VM-signature expression profiling of non-TNBC breast cancer cell lines. Figure S2. In silico regulon-enrichment analysis prioritizes EMT- and stem-cell-associated transcription factors as candidate regulators of the VM-competent state in TNBC. Figure S3. Hypoxia is associated with the vasculogenic mimicry transcriptional program in breast cancer and in TNBC cell lines. Table S1. Gene enrichment for various signature modules from DepMap. Video S1. Live imaging of 3B-11 mouse endothelial cells (angiogenic model) undergoing in vitro tube formation in 3D culture in vitro. Video S2. Time-lapse imaging of MDA-MB-468 (TNBC) showing absence of tube formation (VM) in 3D culture. Video S3. Time-lapse imaging demonstrating tube-forming capability (VM) of MDA-MB-231 (TNBC) in in vitro 3D culture. Video S4. Live imaging showing VM-like tube formation by MDA-MB-231-4175 (lung tropic TTNBC) in vitro 3D culture.
