# Supplementary material for: Distinct Transcriptional and Migratory Programs Are Associated with Vasculogenic Mimicry Heterogeneity in Triple-Negative Breast Cancer
Source: Cancers (Basel). 2026 May 29;18(11):1789. doi: 10.3390/cancers18111789 (PMC13256714; doi:10.3390/cancers18111789)
Supplement: Supplementary file 1 [file cancers-18-01789-s001.zip › Supplementary Figure S1.pdf]

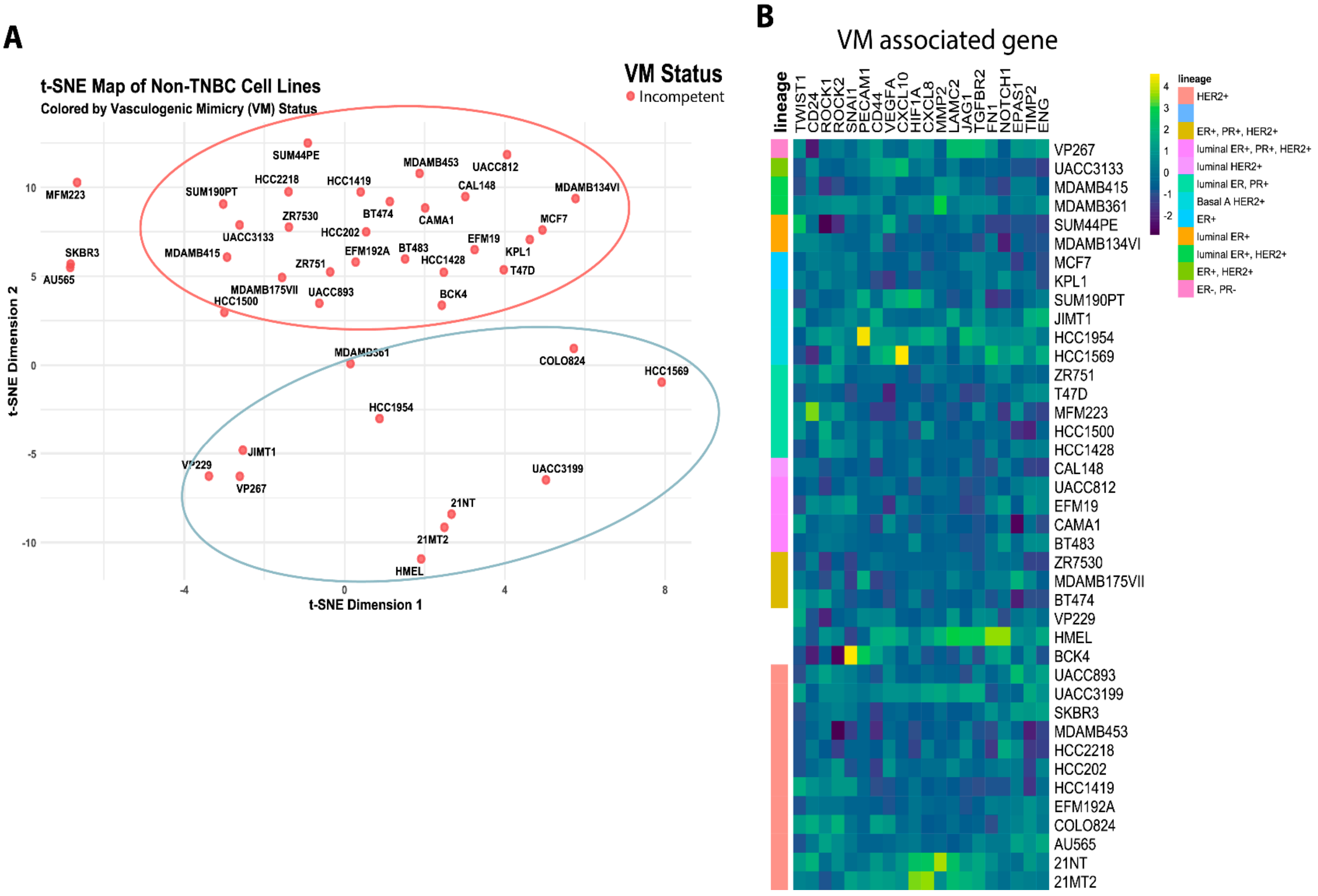

Supplementary Figure S1. Transcriptional clustering and VM-signature expression profiling of non-TNBC breast cancer cell lines.

(A) t-SNE projection of non-TNBC breast cancer cell lines based on transcriptomic profiles, with points colored by molecular subtype. (B) Heatmap of curated VM-associated gene expression (z-scored across the panel) across the non-TNBC cell lines shown in A, illustrating heterogeneous enrichment with a minority of lines (e.g., HCC1569, HCC1954, HMEL, JIMT1) displaying modest VM-signature activity.
