# Supplementary material for: Distinct Transcriptional and Migratory Programs Are Associated with Vasculogenic Mimicry Heterogeneity in Triple-Negative Breast Cancer
Source: Cancers (Basel). 2026 May 29;18(11):1789. doi: 10.3390/cancers18111789 (PMC13256714; doi:10.3390/cancers18111789)
Supplement: Supplementary file 1 [file cancers-18-01789-s001.zip › Supplementary figure S2.pdf]

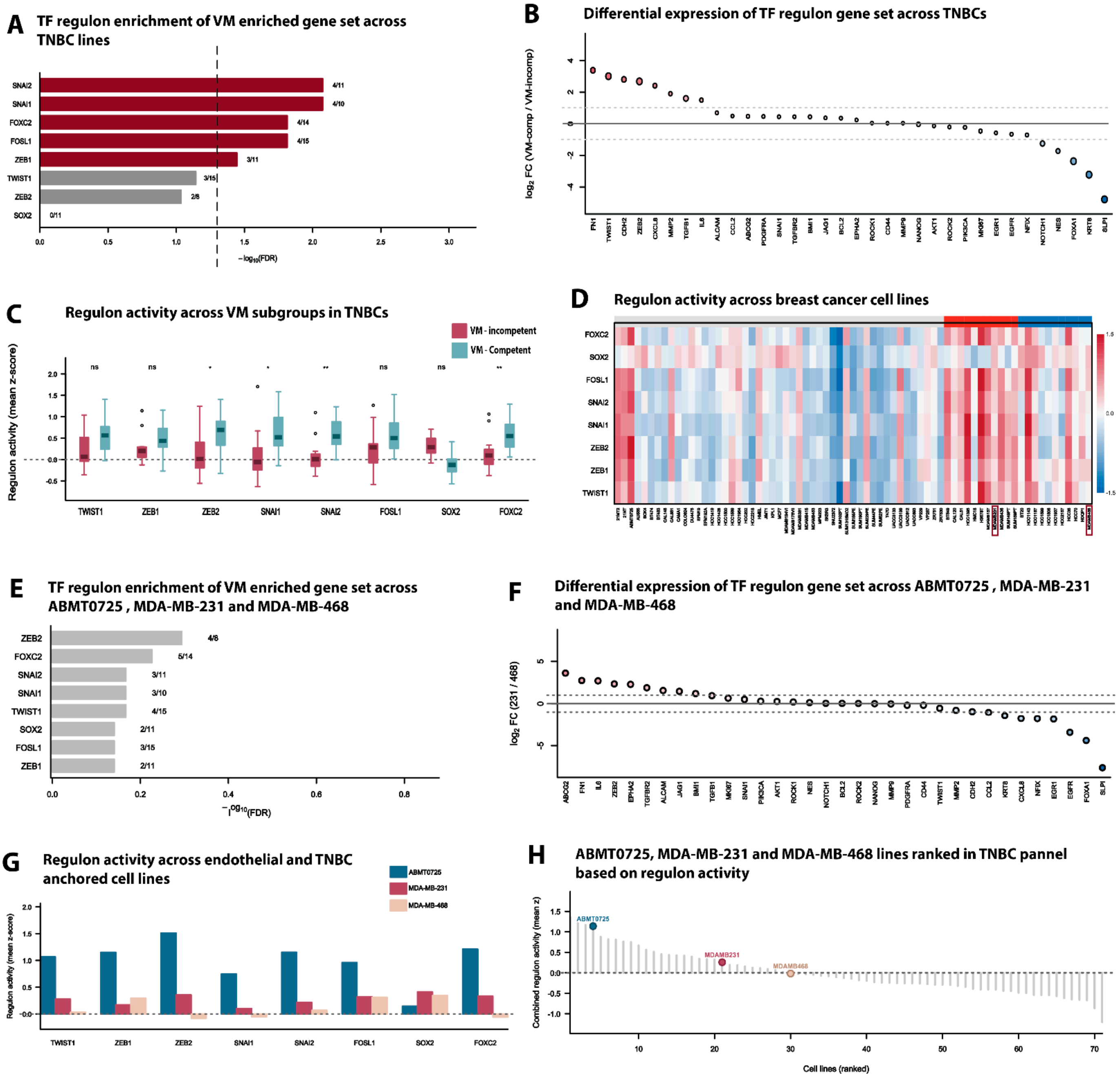

Figure S2. In silico regulon-enrichment analysis prioritizes EMT- and stem-cell-associated transcription factors as candidate regulators of the VM-competent state in TNBC. For each of eight transcription factors (*TWIST1, ZEB1, ZEB2, SNAI1, SNAI2, FOSL1, SOX2, FOXC2*), literature-curated direct target sets were tested for enrichment among genes up-regulated in VM-competent versus VM-incompetent breast cancer cell lines (DepMap), and per-line regulon activity was computed as the mean z-score of target expression across the 71-line panel. (A–D) Panel-level analysis (n = 22 TNBC lines). (A) Fisher's exact enrichment of each TF's target set within the VM-up gene set ($\log_2$FC > 0.5, p < 0.05, Welch's t-test); bars, $-\log_{10}$(FDR), BH-corrected; numbers, overlap / regulon size; red, FDR < 0.05. (B) Log_2 fold-change of regulon target genes between

VM-competent and VM-incompetent lines (red, higher; blue, lower). (C) Regulon activity per cell line, by subgroup; one-sided Mann–Whitney *U* test (ns; *, $p < 0.05$; **, $p < 0.01$). (D) Heatmap of regulon activity for the eight TFs across the 71 breast cancer cell lines (top bar: VM-competent, red; VM-incompetent, blue; other, grey; scale ± 1.5). (E–H) Anchor-line analysis: ABMT0725 (endothelial reference), MDA-MB-231 (VM-competent), MDA-MB-468 (VM-incompetent). (E) Enrichment of each TF's target set within genes up-regulated in MDA-MB-231 versus MDA-MB-468 ($log_2FC > 1$); with one cell line per group no statistical test is applied, and the ranking is presented as a candidate shortlist for follow-up. (F) Log_2 fold-change of regulon members (MDA-MB-231 / MDA-MB-468); colours as in B. (G) Regulon activity for the three anchor lines. (H) Combined regulon activity (mean z-score across top-ranked TFs from E) for all 71 cell lines, ranked from highest to lowest, with anchor lines highlighted.
