# Supplementary material for: Distinct Transcriptional and Migratory Programs Are Associated with Vasculogenic Mimicry Heterogeneity in Triple-Negative Breast Cancer
Source: Cancers (Basel). 2026 May 29;18(11):1789. doi: 10.3390/cancers18111789 (PMC13256714; doi:10.3390/cancers18111789)
Supplement: Supplementary file 1 [file cancers-18-01789-s001.zip › Supplementary Figure S3.pdf]

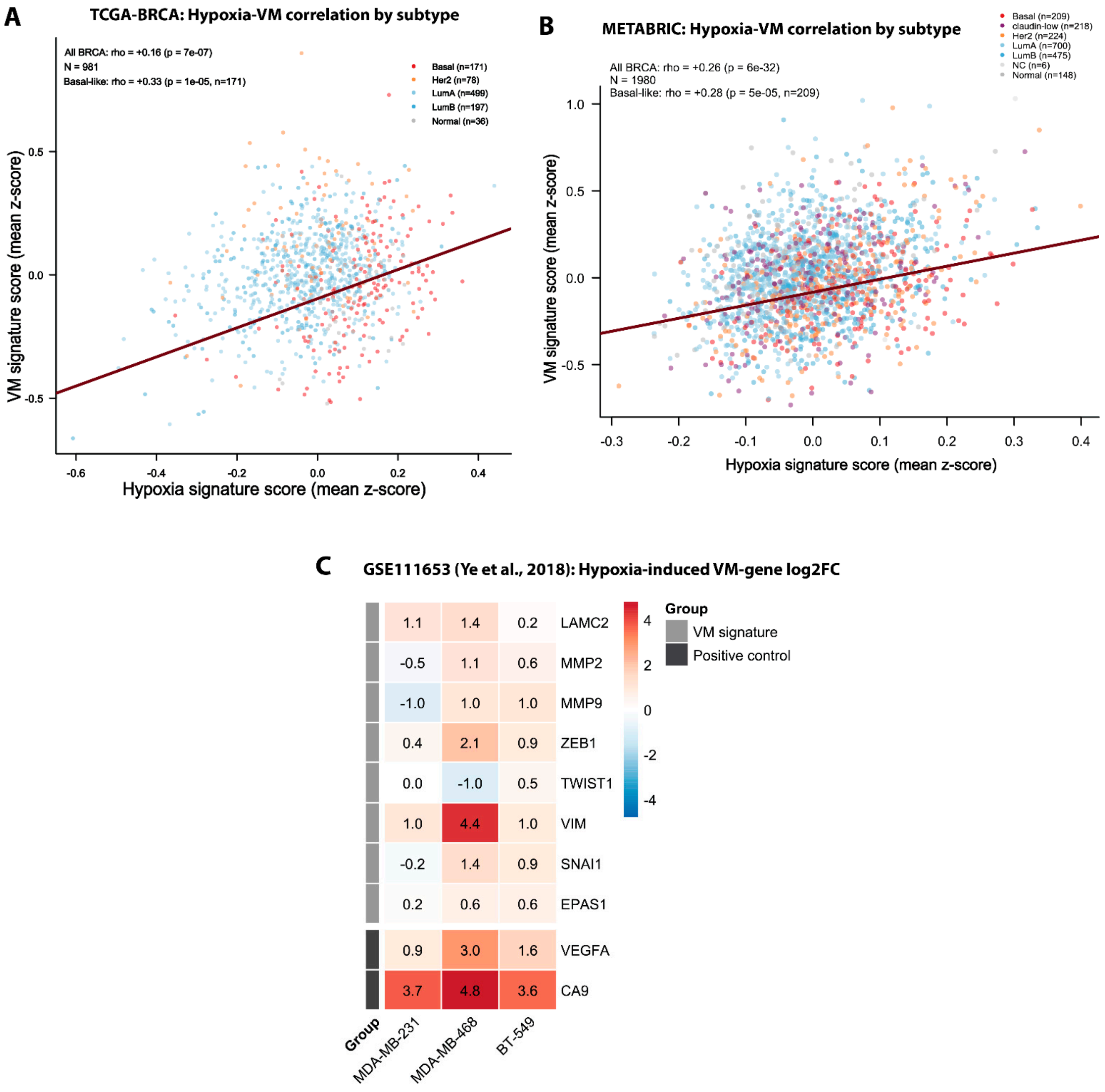

Supplementary Figure S3. Hypoxia is associated with the vasculogenic mimicry transcriptional program in breast cancer and in TNBC cell lines. (A,B) Hypoxia and VM signature scores correlate positively in (A) TCGA-BRCA (*n* = 981) and (B) METABRIC (*n* = 1,980). Scores were derived from the MSigDB HALLMARK_HYPOXIA gene set and a 19-gene VM panel (*CDH5, EPHA2, KDR, VEGFA, LAMC2, MMP14, MMP2, MMP9, COL4A1, COL4A2, ITGAV, ITGB3, ITGB1, CAV1, HIF1A, EPAS1, STAT3, NOTCH1, TWIST1*); see Methods. Each

point is one tumor, colored by intrinsic molecular subtype; the regression line is fit to Basal samples. Spearman correlation is significant in Basal tumors (TCGA: $\rho = +0.33$, $p = 1 \times 10^{-5}$; METABRIC: $\rho = +0.28$, $p = 5 \times 10^{-5}$) and across all samples (TCGA: $\rho = +0.16$, $p = 7 \times 10^{-7}$; METABRIC: $\rho = +0.26$, $p = 6 \times 10^{-32}$).

(C) Hypoxia-induced $\log_2$ fold-change of VM-program genes (top: *LAMC2, MMP2, MMP9, ZEB1, TWIST1, VIM, SNAI1, EPAS1*) and HIF-target positive controls (bottom: *VEGFA, CA9*) in MDA-MB-231, MDA-MB-468, and BT-549 cells from GSE111653 (Ye et al., 2018; 1% vs 20% O_2, 24 h). MDA-MB-468 shows the strongest VM-program induction; robust *CA9* induction confirms intact HIF signaling. Color scale: blue–white–red, centered at zero.
